# Evaluation of day care versus inpatient cataract surgery performed at a Jiangsu public Tertiary A hospital

**DOI:** 10.1186/s12886-018-0800-8

**Published:** 2018-06-05

**Authors:** Min Zhuang, Juan Cao, Minglan Cui, Songtao Yuan, Qinghuai Liu, Wen Fan

**Affiliations:** 1 Department of Ophthalmology, The First Affiliated Hospital with Nanjing Medical University, Nanjing, China; 2 Department of Ophthalmology, The Fourth Affiliated Hospital of Nantong University, Yancheng, China

**Keywords:** Day care, Inpatient, Cataract

## Abstract

**Background:**

High cataract incidence and low cataract surgical rate are serious public health problems in China, despite the fact that efficient day care cataract surgery has been implemented in some public Tertiary A hospitals in China. In this study, we compared not only clinical outcomes, hospitalization time and total costs but also payment manners between day care and inpatient procedures for cataract surgery in a Jiangsu public Tertiary A hospital to put forward several instructional suggestions for the improvement of government medical policies.

**Methods:**

In total, 4151 day care cases and 2509 inpatient cases underwent the same cataract surgery in the day care ward and ordinary ward respectively, and were defined as two groups. General information, complications, postoperative best corrected visual acuity (BCVA), hospitalization time, total costs and especially payment method were analyzed to compare day care versus inpatient.

**Results:**

The general data display no significant differences (*P* > 0.05), and no significant difference between complications and postoperative BCVA were observed between the two groups (*P* > 0.05). The period of stay in hospital was significantly different (*P* < 0.001). The total costs were lower for day care than for inpatients (*P* < 0.001). To avoid sampling error, we analyzed the data of payment manner for each patient among this period. Day care patients tended to pay for the procedure using the Urban Employees Basic Medical Insurance (UEBMI) method, while inpatients tended to use the Out-of-Pocket Medical Treatment (OMT) payment method (*P* < 0.001).

**Conclusion:**

Day surgery of cataract is more cost-effective and efficient than inpatient surgery with equivalent clinical outcomes. As an efficient therapeutic regimen, day care surgery should be further promoted and supported by the government policies.

## Background

A cataract is an opacity of the lens in the eyes, which can lead to blurry vision or blindness. Cataracts may be classified into three categories: age-related cataracts, metabolic cataracts, and cataracts secondary to other causes. Age-related cataracts are the most common type in adults [1]. With a rapidly growing and aging population, the incidence of cataracts is subsequently increasing. WHO estimated that there were 95 million people visually impaired due to cataracts in 2014. According to the former therapeutic regimen, patients prefer being hospitalized for several days for cataract surgery, while the concept of day care cataract surgery is currently increasingly introduced [2]. In the U.S. and Europe and America over 60% cases of cataract surgeries are carried out as day care surgeries. Day surgery, a fast and safe therapeutic regimen [3], is a surgical procedure wherein hospitalization, surgery and discharge occur within 24 h after a short post-operative recovery. Ophthalmologic operations are short and quick, making them suitable for day surgery, which allows for a more effective use of medical resources and reduction of average hospitalization days. It has been shown that inpatient care for age-related cataract surgery may not only be cheaper, but also as effective as inpatient treatment after the same surgery [4].

Cataracts are the leading cause of blindness and the second-leading cause of vision loss in China [5]. With the promotion of “Vision 2020-the Right to Sight” propaganda, the rate of cataract surgury has increased in China in recent years [6]. In the past ten years, it has made great progress in blindness prevention and treatment. In 2005, the number of cataract extractions was only 572,000, at the rate of 440 cases per one million people, per year. By 2014, the number of cataract extractions reached 1.9 million cases, a rate of 1400 cases per one million people, per year [7]. Despite this, some patients continue to avoid or refuse cataract surgery due to the costs and fear of surgery [8]. Until now, the high incidence of cataracts and low surgical rate is still a serious public health problem. It is essential to develop day surgery cataract treatment programs due to the fact that they use fewer hospital resources, are cheaper, and more efficient than those procedures which result in ordinary hospitalization.

In 2011, the International Association for Ambulatory Surgery (IAAS) defined day surgery in the UK and Ireland as: the patient must be admitted and discharged on the same day, with day surgery as the intended management [9]. The rate of day care cataract surgery is almost 100% in Denmark and nearly 0% in Austria [10]. In China, day surgery has been implemented only in recent years, later than other countries, but has been implemented in Hong Kong since the 1990s [11]. China joined the IAAS officially in May 2013, as the 22nd member. By October 2014, 28 municipal hospitals performed day surgeries, and from January to October 2014, the daily proportion of day surgeries of the top 10 hospitals accounted for 76.9% of the municipal hospitals in Shanghai [12]. Today, day surgery of cataracts is more widely accepted morethan previously in China [13, 14]. The day care cataract surgery has been implemented by the Ophthalmology of the First Affiliated Hospital of Nanjing Medical University since August 1, 2014, which was the first hospital implementing day surgery in Jiangsu Province.

China launched a government-run mandatory insurance program, the Urban Employee Basic Medical Insurance (UEBMI) at the end of 1998. Current and former employees of urban enterprises and institutions are insured with UEBMI. Rural populations are mainly insured by New Rural Cooperative Medical Care. While the other medical insurances include Urban Residents Basic Medical Insurance (URBMI), Poverty Salvation Free Medical Care, Free medical care. Meanwhile, some citizens pay for their medical care out-of-pocket or are covered by other medical insurances.

In this study, we compared the differences between day care and inpatient cataract surgery, the results of which will guide the further application of day care or inpatient care following cataract surgery.

## Methods

Data of patients who underwent cataract operations was pulled from the cataract operation HIS database of the First Affiliated Hospital of Nanjing Medical University from August 1, 2014 to December 31, 2016. There are no absolute contraindications for day surgery patients. Patients with stable chronic medical conditions are considered to be suitable for day surgery [15]. At the First Affiliated Hospital of Nanjing Medical University, patients chose day care or inpatient cataract surgery on a voluntary basis. During this period, all fee standards remained the same, and there was no day care patient transferred to the ordinary ward due to severe surgical complications. In this retrospective cohort study, inclusion and exclusion criteria included: 1) Patients were at least age thirty with a stable state of health and were diagnosed with cataracts in one or both eyes; 2) Patients with other ocular comorbidities that could affect the postoperative prognosis was ruled out by fundus assessment; 3) Each patient underwent surgery for only one eye; 4) All the patients were operated upon under topical anesthesia and were performed cataract surgery by phacoemulsification combined with posterior chamber intraocular lens implantation.These two groups were respectively termed as day care group and inpatient group. The surgeons in the two groups were the same, all of whom were senior doctors with rich clinical experience, performing over 5000 cases of phacoemulsification.The surgical processesfollowed standard procedures [1]. The length from the patient’s registration to check-out was called hospitalization time, and less than a day was counted as one day. The costs consisted of the operation treatment fee, cost of the intraocular lens, anesthesia fee, preoperative inspection fee,cost of drugs, and expense of nursing care and hospital bed. All the patients had ophthalmic examinations including BCVA testing, slit-lamp examination, and intraocular pressure at one day, one week, one month, and three months after surgery. BCVA was examined on the basis of standard logarithmic visual acuity chart (GB11533–2011).

Based on these two groups, we analyzed differences of gender, age, and preoperative Best Corrected Visual Acuity (BCVA) between the two groups. Intraoperative posterior capsule rupture was also analyzed. Postoperative complications including cornea edema and intraocular hypertension the first day after surgery, and postoperative BCVA one month after surgery were analyzed. Meanwhile the differences of the hospitalization time, total costs and payment manners between the two groups were also analyzed. All statistical analyses were assessed using a t-test and χ2 test by IBM SPSS Statistics 24, and *P* < 0.05 was considered to be statistically significant.

## Results

### General information

A total of 2809 males (1724 in the day care group and 1085 in the inpatient group) and 3851 females (2427 in the day care group and 1424 in the inpatient group) patients were included in the current study. The average age was 70.54 ± 11.5 in the day care group and 70.01 ± 12.59 in the inpatient group. The average preoperative BCVA was 3.82 ± 0.48 in the day care group and 3.80 ± 0.50 in the inpatient group. Generally, there were no significant differences in gender (*P* = 0.170), age (*P* = 0.150), or preoperative visual acuity (*P* = 0.062) between day care patients and inpatients (Table 1).

**Table 1:** General information before the surgery

Information		Day care (total *n* = 4151)	Inpatient (total *n* = 2509)
Gender	Male	1724	1085
	Female	2427	1424
Age		70.54 ± 11.51	70.01 ± 12.59
Preoperative BCVA		3.82 ± 0.48	3.80 ± 0.50

The data of gender were presented as number of participants (%); the data of age and preoperative BCVA were presented as average ± SD

### Complications and outcomes

Posterior capsule rupture (PCR) as a intraoperative complication (*P* = 0.715) was noted in 291 cases in the day care group and in 170 cases in the inpatient group (Table 2). The main postoperative complications such as cornea edema (*P* = 0.973) and intraocular hypertension (*P* = 0.569) occurring the first day after surgery were noted in both groups (Table 2). The average postoperative BCVA was 4.84 ± 0.18 in the day care group and 4.86 ± 0.84 in the inpatient group, with no significant difference (*P* = 0.290) between the two groups (Table 3). As aforementioned, there were no significant differences in intraoperative complications, postoperative complications, and postoperative BCVA between the two groups.

**Table 2:** Intraoperative and postoperative complications

Complications		Number of participants (%)		*P*
		Day care (total *n* = 4151)	Inpatient (total *n* = 2509)	
	+	291 (7.0%)	170 (6.8%)	
Posterior capsule rupture				0.715
	–	3860 (93.0%)	2339 (93.2%)	
	+	466(11.2%)	281 (11.2%)	
Cornea edema				0.973
	–	3685 (88.8%)	2228 (88.8%)	
	+	323 (7.8%)	205 (8.2%)	
Intraocular hypertension				0.569
	–	3828 (92.2%)	2304 (91.2%)	

*n* number of participants; + numbers of participants having complications; − numbers of participants not having complications; *P* > 0.05 shows no significant difference

**Table 3:** General information after the surgery

Information	Average ± SD		*P*
	Day care (total *n* = 4151)	Inpatient (total *n* = 2509)	
Postoperative BCVA	4.84 ± 0.18	4.86 ± 0.84	0.290
Hospitalization time (days)	1 ± 0	4.40 ± 1.69	< 0.001
Costs (¥)	6893.68 ± 1362.89	7849.70 ± 1432.70	< 0.001

Total *n* = 1000; *P* > 0.05 shows no significant difference

### Hospitalization time and costs

The average hospitalization time was 1 ± 0 days for the day care group and 4.40 ± 1.69 days for the inpatient group. Those patients who chose the day care option for cataract surgery were hospitalized for a much shorter time (*P* < 0.001) than those who chose the inpatient procedure (Table 3). The average costs paid by patients in the day care group were ¥6893.68 ± 1362.89. While the average costs paid by inpatients were ¥7849.70 ± 1432.70, the total costs paid by patients in the day care group were less (*P* < 0.001) than those in inpatient group (Table 3). In conclusion, day surgery of cataracts is more efficient and cost-effective than inpatient surgery.

### Medical insurances

To minimize sampling error, the data of payment method for each patient who underwentcataract surgery within two years was analyzed. There were over six methods with which to pay for total costs of the medical care. Two main methods were Urban Employees Basic Medical Insurance (UEBMI) and Out-of-pocket Medical Care (OMC) (details in Table 4). There was a significant difference in these main payment methods between these two groups (*P* < 0.05).

**Table 4:** Different payment manners for surgery expenses

Payment manners	Number of participants (%)		*P*
	Day care (total *n* = 4151)	Inpatient (total *n* = 2509)	
Urban employees basis medical insurance	2029 (48.9%)	765 (30.5%)	< 0.001
Urban residents basic medical insurance	73 (1.8%)	70 (2.8%)	0.006
New rural cooperative medical care	3 (0.1%)	3 (0.1%)	0.533
Poverty salvation	0 (0%)	2 (0.1%)	0.069
Free medical care	197 (4.7%)	102 (4.1%)	0.214
Out-of-pocket medical care	1768 (42.6%)	1529 (60.9%)	< 0.001
Others	81 (2.0%)	38 (1.5%)	0.200

*n* number of participants; *P* > 0.05 shows no significant difference

## Discussion

In our study, the clinical outcomes exhibited by the patients of the day care group were nearly equivalent to those of the inpatient group, underscoring the safety and effectivity of cataract day surgery. The results also demonstrated that day care surgery shortened the patient’s hospitalization time, which according to the patients’ satisfaction investigations, resulted in a reduction of nervousness, anxiety, and mental stress caused by the long-term hospital stay subsequently reducing the operation burden and improving patient satisfaction. Before the cataract surgery, patients were typically required to undergo general examinations and tests, the expenses of which were included in the total costs paid by inpatients and not included in the total costs paid by day surgery patients. As these expenses were a small part of the total cost, they were not the leading cause of the the difference in cost between day care and intpatient procedures. At the same time, hospitalization costs including ward bed expenses, nursing expenses, and treatment expenses were significantly reduced due to the shortened hospitalization time. Day surgery patients were discharged from hospital within 24 h, which had the dual benefit of greatly reducing the total hospitalization costs and accelerating the turnover of hospital beds, leaving more hospital beds available for severe cases and subsequently greatly increasing the efficiency of bed utilization.

UEBMI was the first basic medical insurance established during medical security system reform in China. With the principle of “wide coverage and low level”, it provides basic medical insurance for urban workers by combining government subsidies and medical insurance premiums payed by individuals and enterprises together (called “social pooling combined with personal accounts”). [16]. The government has also launched a project called Poverty Salvation For The Poor. Free medical service refers to a social security system implemented by China to cover state staff and offers free medical treatment and preventive service provided by the medical and health departments according to the regulations. Currently, one of the biggest bottlenecks in the development of day surgery in China is the inability to manage the reimbursement. Some studies showed that the reimbursement level of medical insurance was an important factor influencing patients’ choice of day surgery [17]. In many areas of China, day care procedures are not reimbursed by the hospital, or only the expenses for the operation can be reimbursed, excluding the preoperative inspection expenses. Although some terms have already been piloted, there is still no national standard for payment, limiting the development of day surgeries in China. Patients in this study mostly chose two methods (UEBMI and OMC) to pay the expenses, and patients who chose UEBMI in the day care group were more than those in inpatient group, mainly because local residents with UEBMI preferred day care due to the aforementioned advantages. Patients who chose OMC in the inpatient group were more than those in the day care group. For example, most non-Nanjing natives who had other medical insurances at their registered permanent residence, chose inpatient for cataract surgery because they had to pay the medical costs through OMC and apply for reimbursement back to the origin residence. Day care medical expenses not being reimbursed may be the reason why more OMC patients chose regular hospitalization and not day care procedures.

In order to improve day surgery, the Guidelines on Comprehensive Reform Pilot of Urban Public Hospitals announced by General Office of the State Council in May 2015 (No.38 [2015] of the General Office of the State Council) clarified that diagnosis and therapy technologies including day surgery should be gradually brought into the scope of medical insurance payments on the basis of normalizing day surgeries. In 2015, 20 day surgeries including age-related cataract were brought into single diseases in Jiangsu province. Twenty-three day surgeries have been paid according to medical insurance since 2016, with the highest reimbursement rate reaching 85% in Jiangxi province [18]. The Guidelines on Comprehensive Reform Pilot of Urban Public Hospitals announced by General Office of the State Council on June 28, 2017 (No.55 [2017] of the General Office of the State Council) clarifies the importance of medical insurance payments for day surgeries again. In this study, we didn’t distinguish the costs of differingartificial lenses, which made the results less rigorous. In further studies, this could be improved by a larger sample size.

In conclusion, our results confirm that cataract day surgery is high-quality and low-cost. Medical insurance payments for day surgery is need urgent improvement. Further optimization and reorganization of the medical service process is essential for day care wards of hospitals to form a high efficiency management and operation system, making it convenient for patients to seek medical treatment and reducing the economic burden patients experience. Along with the rapid economic development in our country, the organization and effective use of medical resources is an urgent and immediate concern.

## Conclusions

In this study, we compared the differences between day care and inpatient cataract surgery, the results of which confirm that day care cataract surgery is high-quality and low-cost. Given these results, it is recommendable that the practice of day care cataract surgery be expanded in China.

## Abbreviations

BCVA: Best Corrected Visual Acuity
IAAS: International Association for Ambulatory Surgery
OMT: Out-of-Pocket Medical Treatment
PCR: Posterior capsule rupture
UEBMI: Urban Employees Basic Medical Insurance
URBMI: Urban residents basic medical insurance
